# Phytohormone-Mediated Molecular Mechanisms Involving Multiple Genes and QTL Govern Grain Number in Rice

**DOI:** 10.3389/fgene.2020.586462

**Published:** 2020-11-12

**Authors:** Priyanka Deveshwar, Ankita Prusty, Shivam Sharma, Akhilesh K. Tyagi

**Affiliations:** Interdisciplinary Centre for Plant Genomics and Department of Plant Molecular Biology, University of Delhi, New Delhi, India

**Keywords:** grain number, panicle branching, phytohormones, rice, tiller, yield

## Abstract

Increasing the grain number is the most direct route toward enhancing the grain yield in cereals. In rice, grain number can be amplified through increasing the shoot branching (tillering), panicle branching, panicle length, and seed set percentage. Phytohormones have been conclusively shown to control the above characteristics by regulating molecular factors and their cross-interactions. The dynamic equilibrium of cytokinin levels in both shoot and inflorescence meristems, maintained by the regulation of its biosynthesis, activation, and degradation, determines the tillering and panicle branching, respectively. Auxins and gibberellins are known broadly to repress the axillary meristems, while jasmonic acid is implicated in the determination of reproductive meristem formation. The balance of auxin, gibberellin, and cytokinin determines meristematic activities in the inflorescence. Strigolactones have been shown to repress the shoot branching but seem to regulate panicle branching positively. Ethylene, brassinosteroids, and gibberellins regulate spikelet abortion and grain filling. Further studies on the optimization of endogenous hormonal levels can help in the expansion of the grain yield potential of rice. This review focuses on the molecular machinery, involving several genes and quantitative trait loci (QTL), operational in the plant that governs hormonal control and, in turn, gets governed by the hormones to regulate grain number and yield in rice.

## Introduction

The world’s population is predicted to crest over 9 billion by 2050, with the current growth rate. Changes in the patterns of consumption and climate demand a holistic approach to increase food production and achieve global food security (Stamm et al., 2011; Cole et al., 2018; Tyczewska et al., 2018). Rice is the largest contributor to a daily per capita average calorie consumption (19%) in the world. Although current global rice production is in surplus, increasing population growth poses a threat to adequate supplies in the future (Elert, 2014). Hence, rice grain yield should be increased to fulfill future demands, and various strategies have been suggested to increase the yield potential (Khush, 2013). Grain number is a major trait of grain yield in rice and is a direct function of the number of productive tillers, numbers of panicles per plant, and panicle branching. Other traits like panicle size or panicle length and effective seed set also contribute to the grain number and hence grain yield in rice. Panicle length determines the number of primary branches, whereas the length of the panicle branch determines the compactness of the inflorescence. The seed set rate is governed by grain filling and the ratio of seed abortion. In rice, many genes and quantitative trait loci (QTL) have been identified that determine the grain yield and are an indispensable genetic resource for crop improvement efforts worldwide (Jeon et al., 2011; Sreenivasulu and Schnurbusch, 2012; Gouda et al., 2020; Sakuma and Schnurbusch, 2020).

In crop plants, the branching pattern of lateral organs plays a central role in defining the plant architecture and the crop yield (Wang and Li, 2005). Shoot branches in rice produce tillers at the vegetative stage and panicles at the reproductive stage (McSteen and Leyser, 2005). Panicles are highly branched inflorescences that are produced on the tillers, and multiple axillary meristems initiate their development. The final panicle architecture is pre-established during development at the meristematic zones that define the branching arrangement and positioning of spikelets. The branch meristem produces primary and secondary branches, spikelet meristem forms spikelets, and floral meristem is responsible for floret and floral organ development. Phytohormones are the major internal factors that regulate these developmental events (Barazesh and McSteen, 2008; McSteen, 2009).

Phytohormones are small regulatory molecules that form an elaborate and sophisticated regulatory network in coordinating various developmental aspects of yield-related traits and thus control the yield potential of the plant (Zhang and Yuan, 2014). Auxins play a role in axillary meristem initiation, while cytokinins are involved in regulating meristem size and activity and thus affect panicle branching indirectly. Besides, cytokinin also promotes axillary bud outgrowth, whereas auxins and strigolactones inhibit axillary bud growth and affect panicle development (Ferguson and Beveridge, 2009; Shimizu-Sato et al., 2009; Dun et al., 2012). Cytokinins, auxins, and jasmonic acid are also involved in meristem fate determination (Zhang and Yuan, 2014). Other hormones like brassinosteroids, gibberellins, and ethylene are also involved in defining the features of panicle morphology and seed set. Gibberellins and brassinosteroids have been shown to regulate the spikelet abortion in addition to shoot branching (Ali et al., 2019). Ethylene is revealed to regulate grain filling and contribute to an effective seed set (Ali et al., 2019). Studies suggest that an intricate network of hormonal pathways regulates the panicle development and modulates panicle architecture (Zhang and Yuan, 2014).

Balancing panicle-related traits such as panicle length, panicle number, and the numbers of primary and secondary branches per panicle are key to improving the number of grains per plant. These traits can be manipulated by fine calibration of molecular component of hormonal signaling to enhance the grain yield efficiently. Thus, the information regarding the hormonal pathways, their homeostasis, and their complex networks is instrumental in designing high yielding crops for the future. The current review provides an overview of the recent progress of the genetics of phytohormone actions and their crosstalks in the context of grain number as a complex agronomic trait in rice.

## Control of Grain Number by Phytohormones

Auxins, cytokinins, and strigolactones have been convincingly shown to have roles in shoot and panicle branching and also communicate among them to regulate it (Shimizu-Sato et al., 2009; Dun et al., 2012; Zha et al., 2019). Ethylene, brassinosteroids, and auxins interact to regulate panicle differentiation and degeneration (Ali et al., 2019). The role of each hormone in the regulation of grain number trait in rice will be discussed below. Recent elaborated studies that establish various cross-hormonal interactions involved in regulating grain number in rice are discussed in a separate section. Table 1 is a compilation of major studies done so far to characterize genes involved in hormonal regulation of grain number.

**TABLE 1:** Some genes/quantitative trait loci (QTL) regulating the grain number per plant in rice.

Gene/QTL	RGAP Locus ID	Hormone involved/affected	Nature of protein encoded	Phenotype obtained for	Phenotype related to grain yield	References
***AFB6***	LOC_Os03g08850	Auxin and CK	Auxin receptor	Overexpression	Increase in spikelets per panicle and primary branch number; high photoperiodic sensitivity; delayed heading	He et al., 2018
***ASP1***	LOC_Os08g06480	Auxin	TOPLESS-related transcriptional co-repressor	Recessive mutant	Aberrations in spikelet morphology and branching pattern; reduction in rachis length and spikelet number with multiple phenotypes	Yoshida et al., 2012
***BG1***	LOC_Os03g07920	Auxin	Novel membrane localized protein	Dominant mutant/overexpression	Increase in grain size, panicle size, plant height along with other pleiotropic phenotypes	Liu L. et al., 2015
***CYP71D8L***	LOC_Os02g09220	GA	Cytochrome P450 monooxygenase	Gain-of-function mutant and overexpression	Reduced panicle length, grain number per panicle, and reduced plant height	Zhou et al., 2020
***D2, SMG11***	LOC_Os01g10040	BR	Cytochrome P450 (CYP90D2)	Recessive mutant	Dense and erect panicles; increase in secondary branching and number of grains per panicle; small grains	Fang et al., 2016
***D3***	LOC_Os06g06050	SL	F-box protein	Loss-of-function mutant	Reduction in panicle size and plant height; higher tillering	Ishikawa et al., 2005
***D4***	LOC_Os03g12660	BR	Cytochrome P450 (CYP90B2)	Overexpression	Restores clustered panicle branching phenotype in *pmm1-1* background	Li et al., 2018
***D10***	LOC_Os01g54270	SL	CCD8	Loss-of-function mutant	Reduction in panicle size and plant height; higher tillering	Ishikawa et al., 2005; Arite et al., 2007
***D14, HTD2, D88***	LOC_Os03g10620	SL	α/β-fold hydrolase (receptor)	Loss-of-function mutant	Reduction in panicle size and plant height; higher tillering; smaller seed size	Ishikawa et al., 2005; Wang et al., 2017; Liang et al., 2019
***D17, HTD1***	LOC_Os04g46470	SL	Carotenoid cleavage dioxygenase 7 (CCD7)	Loss-of-function mutant	Reduction in panicle size and plant height; higher tillering	Ishikawa et al., 2005; Zou et al., 2006
***D27***	LOC_Os11g37650	SL and Auxin	β-carotene isomerase	Loss-of-function mutant	Reduction in panicle size and plant height; higher tillering	Ishikawa et al., 2005; Hao et al., 2009
***D53***	LOC_Os11g01330	SL	Class I Clp ATPase	Gain-of-function mutant	Reduction in panicle size and plant height; higher tillering	Zhou et al., 2013
***DCL3a***	LOC_Os01g68120	GA and BR	RNase III-class Dicer-like 3	Knockdown	Small panicles, reduction in number of secondary branches, larger flag leaf angle and reduced plant height	Wei et al., 2014
***DEP1***	LOC_Os09g26999	CK	Phosphatidylethanolamine-binding protein like domain protein	Gain-of-function mutant	Increase in number of grains per panicle and reduction in length of inflorescence internode	Huang et al., 2009
***Dof12***	LOC_Os03g07360	BR	Dof transcription factor	Overexpression	Reduction in panicle size, primary branching, secondary branching, grain yield, plant height; aberrant leaf phenotypes	Wu et al., 2015
***DOG, SAP11***	LOC_Os08g39450	GA	A20/AN1 zinc-finger protein	Overexpression	Shorter panicle exsertion; reduced plant height	Liu et al., 2011
***DST***	LOC_Os03g57240	CK	Zinc finger protein	Frameshift mutation	Increased panicle branching, grain number and grain yield	Li et al., 2013
***EATB***	LOC_Os09g28440	Ethylene and GA	AP2/ERF transcription factor	Overexpression	Shortened panicle internodes; increase in the number of panicles per plant and spikelets per panicle; reduced plant height	Qi et al., 2011
***ETR2***	LOC_Os04g08740	Ethylene	Ethylene receptor	Overexpression	Reduction in number of effective panicles and seed-setting rate; erect panicles; delayed flowering and other multiple phenotypes	Hada et al., 2009
***EUI1***	LOC_Os05g40384	GA	Cytochrome P450 monooxygenase	Loss-of-function mutant	Increased panicle exsertion; elongated uppermost internode; taller plants	Luo et al., 2006
***FZP***	LOC_Os07g47330	Ethylene	ERF transcription factor	Mutant	Defect in spikelet development; presence of sequential round of branches instead of florets/spikelets	Komatsu et al., 2003
***Gn1a*, *OsCKX2***	LOC_Os01g10110	CK	Cytokinin oxidase/dehydrogenase	Loss-of-function mutant	Increase in number of grains per panicle and grain yield	Ashikari et al., 2005
***GNP1, GA20ox1***	LOC_Os03g63970	GA and CK	GA20-oxidase 1	Near-isogenic line NIL-*GNP1*^*TQ*^	Increased total grain number per panicle; filled grain number per panicle and secondary branch number; slight increase in plant height, slight decrease in grain length, width and 1,000-grain weight	Wu et al., 2016b
***GSN1*, *OsMKP1***	LOC_Os05g02500	CK and BR	Mitogen-activated protein kinase	Overexpression	Increased number of seeds per panicle and reduced seed size	Guo et al., 2018
***IPA1, WFP, OsSPL14***	LOC_Os08g39890	CK and SL	SQUAMOSA promoter binding protein -like 14	Overexpression	Increase in number of panicle branches and grain yield	Jiao et al., 2010; Miura et al., 2010
***LAX1***	LOC_Os01g61480	Auxin	bHLH transcription factor	Mutant	Abnormal spikelet meristem development and panicle architecture	Komatsu et al., 2001
***LOG***	LOC_Os01g40630	CK	Cytokinin riboside 5′-monophosphate phosphoribohydro-lase	Mutant	Reduction in panicle size and abnormal panicle branching	Kurakawa et al., 2007
***LP, EP3***	LOC_Os02g15950	CK	F-box protein	Recessive mutant	Increased panicle size, panicle branching, grain number, and grain yield	Li et al., 2011
***MADS57***	LOC_Os02g49840	GA and SL	MADS-box transcription factor	Knockdown	Reduced panicle exsertion, internode elongation, and plant height	Chu et al., 2019
***NAL1***	LOC_Os04g52479	Auxin	Novel protein	Overexpression	Increased panicle branching and spikelets number per panicle	Fujita et al., 2013
***ONAC096***	LOC_Os07g04560	CK	NAC domain containing protein	T-DNA insertion mutant	Increased tillering, number of panicles, and grain yield	Kang et al., 2019
***NOG1***	LOC_Os01g54860	JA	enoyl-CoA hydratase/isomerase	Overexpression	Enhanced grain number per panicle	Huo et al., 2017
***OsAHP1 and OsAHP2***	LOC_Os08g44350 and LOC_Os09g39400	CK	His-containing phosphotransfer proteins	Knockdown (RNAi)	Reduction in panicle size and seed set	Sun et al., 2014
***OsBZR1***	LOC_Os07g39220	BR and SL	Transcription factor	Overexpression	Promotes tillering	Fang et al., 2020
***OsCCA1***	LOC_Os08g06110	SL	MYB transcription factor	Overexpression	Negatively regulates tillering and positively regulates panicle development	Wang F. et al., 2020
***OsER1***	LOC_Os06g10230	CK and BR	Receptor like kinase	Mutant	Increased number of spikelets per panicle	Guo et al., 2020
***OsJAZ1***	LOC_Os04g55920	JA	Zinc finger protein	Dominant mutant	Defects in spikelet development and morphogenesis	Cai et al., 2014
***OsMED14_1***	LOC_Os08g24400	Auxin	Mediator subunit	Knockdown (RNAi)	Reduced panicle branching, lesser seed set and pleiotropic phenotypes	Malik et al., 2020
***OsPEX5***	LOC_Os08g39080	JA	PTS1 receptor protein	Loss-of-function mutant	Aberrations in spikelet morphology	You et al., 2019
***OsPRR1***	LOC_Os02g40510	SL	Pseudo-Response Regulator	Overexpression	Positively regulates tillering and negatively regulates panicle development	Wang F. et al., 2020
***OsTB1, FC1***	LOC_Os03g49880	SL	TCP family transcription factor	Overexpression	Negatively regulates tillering by inhibiting the outgrowth of axillary buds	Takeda et al., 2003; Minakuchi et al., 2010
***OsVIL2***	LOC_Os12g34850	CK	PHD domain containing protein	Overexpression	Larger panicles, Increased primary and secondary branching and grain number	Yang et al., 2019
***PAY1***	LOC_Os08g31470	Auxin	Peptidase	Dominant mutant	Larger panicles with more secondary branching; increase in grain number per panicle, grain yield per plant, plant height; less number of tillers	Zhao et al., 2015
***PIN1***	LOC_Os02g50960	Auxin	Auxin efflux transporter	Knockout mutant	*pin1cpin1d* double mutant shows pin-like inflorescences with no panicle	Li et al., 2019
***PIN2***	LOC_Os06g44970	Auxin	Auxin efflux transporter	Overexpression	Reduction in panicle length, number of grains per panicle, grain weight per panicle, plant height; increase in tillers angle, number etc.	Chen et al., 2012
***PIN5b***	LOC_Os08g41720	Auxin	Auxin efflux carrier-like protein	Overexpression	Reduction in panicle length, number of seeds per panicle, seed setting rate, tiller number, plant height etc.	Lu et al., 2015
***PMM1, D11***	LOC_Os04g39430	BR	Cytochrome P450 (CYP724B1)	Knockout mutant	Clustered primary branching and small grains	Li et al., 2018
***PRE***	LOC_Os03g55800	JA	Allene oxide synthase	Mutant	Defects in seed setting	Hibara et al., 2016
***Prl5***	LOC_Os05g34854	GA	GA20-oxidase 4	Overexpression	Elongation of panicle rachis and lower primary branches of panicle	Agata et al., 2020
***SP3***	LOC_Os03g55610	CK	Dof transcriptional activator	T-DNA insertion mutant/knockdown mutation	Reduction in plant size, panicle size, number of secondary branches and spikelets	Huang et al., 2019
***TBP1, OsBAK1***	LOC_Os08g07760	BR	SERK protein	Mutant	Increased tillering, panicle branching and seed number but reduced panicle length and seed size	Lin et al., 2017

### Cytokinins

Cytokinins (CKs) are a class of adenine-derived compounds that are categorized as phytohormones involved primarily in cell divisions (Werner et al., 2001; Schaller et al., 2014). Natural CKs can be of two types depending upon the nature of the side chain attached to the adenine moiety. These side chains can be either an aromatic or an isoprene derivative (Sakakibara, 2006). The effect of side chain has been shown to affect shoot growth in *Arabidopsis* (Kiba et al., 2013); however, such effects are not confirmed in other plants. CK homeostasis is maintained in the meristems by a dynamic balance between its biosynthesis, activation, deactivation, reactivation, and degradation (Jameson and Song, 2016).

**FIGURE 1:**
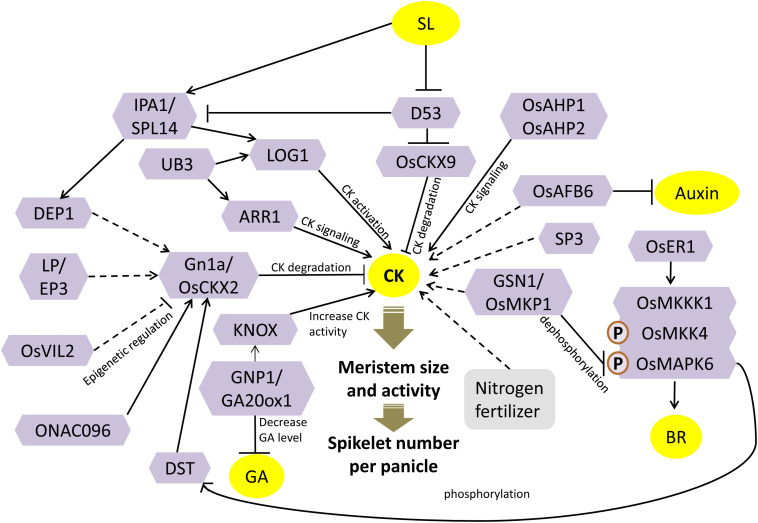
Cytokinin (CK)-dependent meristem activity is central to panicle architecture. Enzymes like OsCKX2, OsCKX9, LOG1, and OsIPTs regulate the concentration of CK. These genes are further regulated by transcription factors, epigenetic regulators, and signaling factors. CK robustly interacts with other hormones, and a complex network determines the inflorescence meristem activity and hence panicle architecture. See the text for details. Solid lines represent direct regulation; dashed lines represent indirect regulation; arrows represent positive regulation; bar-headed lines represent negative regulation; block arrows represent the effect of a response. P represents phosphorylation.

The first important QTL identified in rice for the increase in grain number was *Gn1a*, located on the short arm of chromosome 1 (Ashikari et al., 2005). The *Gn1a* allele of the high-yielding *indica* Habataki variety was held responsible for its 44% more number of grains per panicle compared to Koshihikari *japonica* variety. The *Gn1a* locus encodes for a cytokinin oxidase/dehydrogenase (OsCKX2), an enzyme that degrades the active form of CK irreversibly into adenine or adenosine and the side chains (Figure 1). Its loss-of-function mutation accumulated bioactive CK in the inflorescence meristems. Since CK regulates lateral meristem activity, more number of spikelets are formed that results in increased grain yield (Ashikari et al., 2005). Another QTL for grain number was identified on chromosome 4 and called as *GN4-1*. However, this QTL was fine mapped to a large 190-kb region consisting of 20 genes. Near-isogenic lines (NILs) carrying *GN4-1* also showed increased CK accumulation in young panicles and decreased transcript abundance of as many as eight CKX enzyme family members, including *OsCKX2* (Zhou et al., 2018).

A zinc-finger transcription factor, DROUGHT AND SALT TOLERANCE (DST), has been shown to negatively regulate the CK accumulation in the reproductive meristem by directly regulating the expression of *OsCKX2* (Figure 1). The mutant, *regulator of Gn1a* (*reg1*), was identified as a semidominant allele of *DST* called *DST*^*reg*1^ that showed increased activity in the inflorescence meristem due to higher CK accumulation, resulting in more panicle branching and greater grain yield. The *DST*^*reg*1^ had a frameshift mutation that destroyed its C-terminal EAR motif. Although EAR motif is a well-known repression motif, but here, it showed a non-canonical activation activity, as its deletion destroyed the transcriptional activation activity of DST. Transcript levels of *OsCKX2*, along with other members of the *OsCKX* family, were significantly reduced in the mutant. Chromatin immunoprecipitation (ChIP) assay and electrophoretic mobility shift assay (EMSA) confirmed the binding of DST onto the promoter of *OsCKX2* (Li et al., 2013). DST has been shown to transactivate the OsCKX2 expression and hence positively regulate CK degradation and negatively regulate panicle branching and, consequently, grain number.

The transcription levels of *OsCKX2* were also severely downregulated in the young panicles of mutants of a kelch-repeat containing F-Box protein encoded by *Larger Panicle* (*LP*)/*Erect Panicle 3 (EP3)* (Piao et al., 2009; Li et al., 2011) (Figure 1). LP/EP3 subcellular localization in the endoplasmic reticulum, along with its molecular function as an E3 ubiquitin ligase subunit, proposed its involvement in ER-associated protein degradation. The *lp* mutants showed a significant increase in the size of the panicle, with a considerable increase in grain number and hence an 11% increase in the grain yield. The primary and secondary panicle branches were also significantly increased in the mutant that can be correlated to the *in situ* RNA accumulation of *LP* in the panicle branch primordial region. These phenotypes are similar to the *Gn1a* mutant and thus indicate that perhaps LP positively regulates the expression of *OsCKX2* by the degradation of some unknown proteins. Consequently, LP modulates the levels of CK by possibly indirectly controlling *OsCKX2* gene expression (Li et al., 2011). Although *EP3* and *LP* correspond to the same locus, their mutants showed different phenotypes. The absence of kelch motif in the *ep3* mutant does not increase grain yield; instead, it decreased grain yield and changed the panicle phenotype to a smaller and more compact one (Piao et al., 2009). The role of kelch motif in *LP*/*EP3* needs to be further investigated.

Cytokinin levels in the spikelet meristem are also synchronized by epigenetic regulation of *OsCKX2* gene expression by chromatin-modifying factors. OsVIL2 (*Oryza sativa* VIN3-LIKE 2) is a chromatin-modifying protein that contains a histone binding motif called plant homeodomain (PHD) finger (Yang et al., 2013). Mutation in *OsVIL2* results in smaller plants with few grains per panicle. On the other hand, *OsVIL2* overexpression results in larger panicles with more primary and secondary branches and hence more grain number. The expression of *OsCKX2* was downregulated, whereas the CK levels were increased in *OsVIL2*^*OX*^ plants. Enrichment of OsVIL2 in the transcript initiation region in the promoter of *OsCKX2*, and further enrichment of H3K27me3 around the same promoter region of *OsCKX2*, confirms the epigenetic repression of *OsCKX2* by OsVIL2 (Figure 1). Therefore, OsVIL2 represses *OsCKX2* gene expression by methylating its promoter and thereby increasing the CK accumulation and meristem activity (Yang et al., 2019).

OsCKX2 regulates the grain number by regulating both panicle branching and panicle number (tiller number) (Yeh et al., 2015). Mutation in *ONAC096*, a gene encoding NAC domain-containing transcription factor, results in a 16% increase in grain yield due to a 15% increase in the number of panicles (representation of increased tillering). The mutant *onac096* showed repression of *OsCKX2* gene expression, whereas the *ONAC096*^*OX*^ showed an accumulation of *OsCKX2* transcript. Hence, *ONAC096* negatively regulates the panicle number, tillering, and CK levels by positively regulating *OsCKX2* gene expression (Kang et al., 2019) (Figure 1). Apart from panicle branching and shoot branching, CK has also been implicated in regulating panicle length. *Short Panicle 3* (*SP3*) encodes a DNA binding with one finger (Dof) transcription factor. A knockdown mutation created by T-DNA insertion in the promoter of *SP3* resulted in smaller plants with smaller panicle with significantly lesser number of secondary branches and spikelets. *SP3* is expressed at the branch primordia of the young panicles. The mutant *sp3* had an altered CK homeostasis wherein CK catabolism genes (four members of *OsCKX* gene family) were remarkably upregulated and CK biosynthesis genes (*OsIPT3* and *OsIPT7*) were drastically downregulated, thus resulting in an overall reduction in the CK levels (Huang et al., 2019) (Figure 1).

The activation of CK from its inactive forms, specifically in the meristematic cells, is another crucial metabolic step that regulates the maintenance of meristematic activity. *Lonely Guy* (*LOG*) encodes a novel enzyme called cytokinin riboside 5′-monophosphate phosphoribohydrolase that catalyzes the final step of direct CK bioactivation. It converts CK nucleotides into bioactive free base forms, specifically in the shoot meristem tips. A mutation in it causes a severe reduction in panicle size and abnormal panicle branching due to premature termination of the meristem (Kurakawa et al., 2007). Thus, this localized activation of CK by LOG in the shoot, branch, and spikelet meristems directly regulates grain number (Figure 1).

*IPA1* (*IDEAL PLANT ARCHITECTURE*)/*WFP* (*Wealthy Farmer’s Panicle*) is one of the most promising QTL identified for grain number enhancement (Jiao et al., 2010; Miura et al., 2010). The encoded protein, OsSPL14, is a member of the SQUAMOSA promoter binding protein-like (SPL). OsSPL14 has been shown to directly regulate CK biosynthesis by binding to the promoter of the CK-activating gene, *LOG* (Lu et al., 2013). Additionally, the ortholog of OsSPL14 in maize, UNBRANCHED3 (UB3), that is associated with kernel row number trait, is also shown to bind to the promoter of the rice *LOG* directly and regulates its expression. In addition, UB3 could also bind to the promoters of rice type-A response regulators (*ARRs*) that are regulators of CK signaling (Du et al., 2017). Further, it is known that OsSPL14 directly controls the expression of *DENSE AND ERECT PANICLE 1* (*DEP1*), a major regulator of grain number per panicle, by binding to its promoter (Lu et al., 2013). DEP1 encodes for a phosphatidylethanolamine-binding protein (PEBP)-like domain protein. A gain-of-function mutation, *dep1*, results in increased inflorescence meristem activity, decreased inflorescence internode length, and increased grains per panicle, to produce a dense and erect panicle. The expression of *OsCKX2* was evidently downregulated in NIL-*dep1* (Huang et al., 2009). Thus, *OsSPL14* regulates panicle branching and grain yield by regulating CK levels, by upregulating CK biosynthesis (*LOG* expression), and by downregulating the degradation of CK by positive regulation of *DEP1* and hence downregulation of *OsCKX2* (Figure 1).

The signaling response of CK is mediated by a relay of steps, including subsequent phosphorylation of CK receptors present on the membrane, to the histidine-containing phospho-transfer proteins (AHPs), followed by type-B response regulators in the nucleus. Disruption of CK signaling by simultaneous knockdown of *OsAHP1* and *OsAHP2* via RNA interference (RNAi) in rice plants showed a reduction in panicle size and low seed set along with other pleiotropic effects (Sun et al., 2014) (Figure 1).

Application of nitrogen fertilizers prior to panicle initiation is well known to increase grain yield by increasing the number of spikelets per panicle. Measurement of CK levels after nitrogen fertilizer application showed a local increment of CK levels in the panicles and no change in leaf and root. Analysis of expression profiles of CK metabolism genes highlighted the adenosine phosphate-isopentenyltransferase (*OsIPT*) gene family members that catalyze an initial rate-limiting step of CK biosynthesis. All the *OsIPT* genes showed considerable upregulation in the panicles by nitrogen application. Thus, localized CK accumulation in the panicle lateral meristems is the direct effect of nitrogen fertilizers that increases panicle branching (Ding et al., 2014) (Figure 1).

It seems that the fine tuning of bioactive CKs levels in the inflorescence meristem is a critical trait for engineering panicle architecture and grain number in rice. Figure 1 illustrates the proposed model of CK-mediated regulation of grain number based on the discussed studies. Interactions of CK with other hormones are also depicted in Figure 1, and the details are discussed in section “Crosstalk of Phytohormones to Regulate Grain Number.”

### Auxins

Auxins regulate a plethora of responses both at the cellular and whole-plant level, imparting pleiotropic physiological effects (Thimann and Koepfli, 1935). The developmental module of a panicle involves the transition of the vegetative to the reproductive phase marked by the transformation of shoot apical meristem to axillary meristems and further its fate transition to spikelet meristems (Huijser and Schmid, 2011). Auxin has a pivotal role in panicle development, as it is required for the initiation and maintenance of axillary meristems.

**FIGURE 2:**
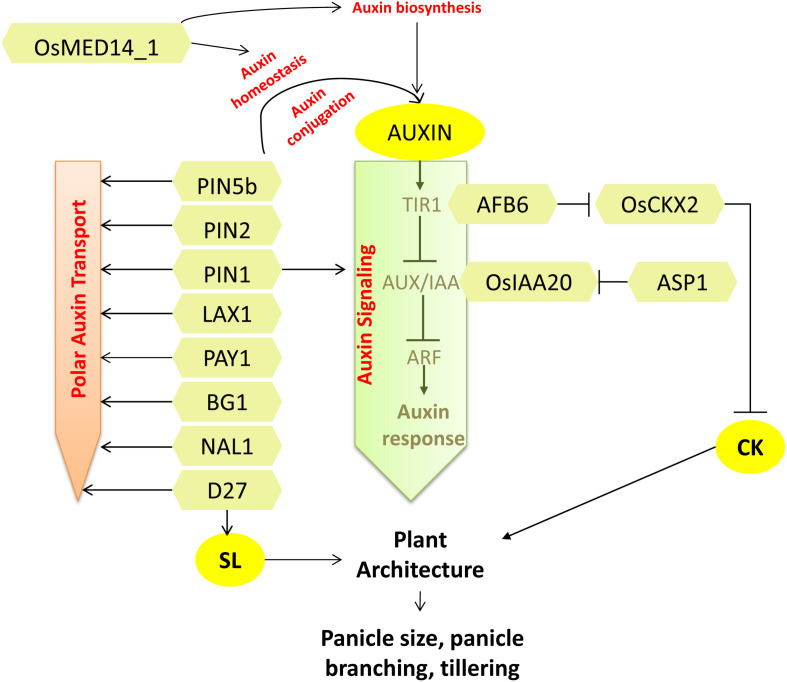
Regulators of auxin signaling regulating plant architecture and affecting grain number. Factors affecting auxin biosynthesis, homeostasis, signaling, and transport identified in rice with functions known in affecting grain number are shown. Interconnections of auxin signaling with CK and SL also contribute to the plant architecture that affects grain number. Arrows represent positive regulation; bar-headed lines represent negative regulation.

Auxin is produced mainly in growing shoot apices and is transported basipetally down the site along specific transport routes through polar transport machinery, and it indirectly inhibits the growth of axillary buds (Sieberer and Leyser, 2006; Zwiewka et al., 2019). Wild rice genotypes have short stature with thin stems, few grains, high tillering, and low yield. A gain-of-function mutation in wild rice introgression line YIL55 changed the plant architecture to that of an ideal crop with increased plant height, lesser number of tillers, thicker stems, larger panicles, and more secondary branches with a remarkable increase in grains per panicle as well as grain yield per plant (38%). The mutation was found in *PLANT ARCHITECTURE AND YIELD 1 (PAY1)* that encodes for a nuclear-localized peptidase and regulates plant architecture by affecting polar auxin transport and altering levels of endogenous indole 3-acetic acid (IAA). The *PAY1* mutation reduced the basipetal transport of IAA (Figure 2). This resulted in a trait wherein apical dominance was enhanced, leading to reduced tiller number. The introduction of the *PAY1* allele in cultivated high-yielding varieties further increased the grain number per panicle and grain yield per plant significantly (Zhao et al., 2015).

*SPIKELET NUMBER* (*SPIKE*) is an allele of *NARROW LEAF 1 (NAL1*) that affects panicle architecture pleiotropically to enhance the grain yield. *NAL1* encodes a novel protein that regulates polar auxin transport (Qi et al., 2008) (Figure 2). The overexpression of *NAL1* results in increased secondary panicle branching and greater number of spikelets per panicle. In addition, the source size and translocation capacity were increased with greater leaf area, robust root system, and vasculature, which contributed to better yield (Fujita et al., 2013).

The establishment of polar auxin transport is brought about by the asymmetrically localized PIN proteins that are instrumental in driving polar cell-to-cell transport of auxin. The PIN-FORMED (PIN) protein family is a group of auxin efflux transporters, having role in catalyzing transport of auxin from cells (Zazímalová et al., 2010). OsPIN5b is an endoplasmic reticulum localized protein that regulates cellular auxin homeostasis by facilitating conjugation-based auxin metabolism (Mravec et al., 2009; Barbez et al., 2012). Overexpression of *OsPIN5b* resulted in pleiotropic phenotypes with overall reduction in growth and more notable reduction in tiller number, biomass, seed setting rate, panicle length, number of seeds per panicle, and, thus, reduction in yield. On the other hand, RNAi plants with reduced expression of *OsPIN5b* presented longer panicles with more seeds. Auxin homeostasis is regulated by auxin conjugation with amino acids and sugars. The ratio of levels of auxin conjugates to that of free IAA was found to be low in *OsPIN5b*^*OX*^ tissues. The enhanced level of free IAA in the overexpression lines positively correlated with higher transcript levels of *OsYUCCA1* (role in IAA biosynthesis). Additionally, overexpression of OsPIN5b disrupted the polar auxin movement and auxin distribution. Conclusively, OsPIN5b is associated with auxin homeostasis, transport, and distribution to modulate plant architecture and plant yield (Lu et al., 2015) (Figure 2). Contrary to *OsPIN5b*, overexpression of another member of PIN family, *OsPIN2*, a functional auxin efflux transporter, showed phenotypes such as short plant height, a greater number of tillers, and larger tiller angle at the vegetative stage. At the reproductive stage, the plants exhibited short panicle length, lesser grains per panicle, lower grain length and breadth, and lower grain weight (Wang et al., 2009; Chen et al., 2012). Predominant auxin accumulation in root–shoot junction depicts its probable role in shoot architecture, but molecular mechanism describing its role in panicle development is yet to be revealed.

A recent report presents the functional divergence of four paralogous genes of *OsPIN1* (Li et al., 2019). Analysis of the mutants generated through CRISPR-Cas9 showed that the *pin1apin1b* double mutant had a role in determining root development, plant height, and tiller angle, whereas the *pin1cpin1d* double mutant developed abnormal, naked, pin-like inflorescences at the flowering stage as exactly shown by *Arabidopsis pin1* mutants (Gälweiler et al., 1998). However, the single mutants did not show any developmental abnormalities. Further investigation at the maturation stage revealed that *pin1cpin1d* mutants completely lost their secondary branches and spikelets, and thus, no panicle was formed. Furthermore, relatively low transcript levels of *OsYUCCAs*, *OsARF*, and *OsIAA* genes in these mutants define the functional role of *OsPIN1* in panicle growth and development through affecting auxin biosynthesis and signaling (Xu et al., 2005; Li et al., 2019) (Figure 2). Knockdown of a Mediator subunit protein encoding gene, *OsMED14_1*, showed various pleiotropic effects, including reduced panicle branching and lesser seed set. Evident alterations of auxin levels and transcript levels of auxin homeostasis genes in the *OsMED14_1*^*RNAi*^ plants suggested the role of auxins in *OsMED14_1*-mediated regulation of grain number (Malik et al., 2020). The *BIG GRAIN 1* (*BG1*) is another gene, which encodes a plasma membrane-localized protein that is shown to be a positive regulator of grain size. Plants with suitable overexpressed levels of *BG1* showed increased plant height, longer leaves, larger panicles, and increased grain size by regulating around 50% increased basipetal auxin distribution and transport (Liu L. et al., 2015) (Figure 2).

*BARREN STALK1* (*BA1*) is a maize gene encoding a non-canonical bHLH transcription factor that is a determinant of inflorescence patterning. *BA1* regulates axillary meristem formation by generating an auxin response maxima via polar auxin transport that flanks the primordial inflorescence meristem (Gallavotti et al., 2004, 2008). *LAX PANICLE 1* (*LAX1*), the rice homolog of *BA1*, is also shown to regulate the initiation and maintenance of the axillary meristem. Accordingly, the *lax1* panicles were highly abnormal with an absolute absence of lateral spikelet but the presence of a terminal spikelet (Komatsu et al., 2001). An auxin maxima is required for both reproductive and vegetative axillary meristem in rice and maize (Oikawa and Kyozuka, 2009) in contrast to *Arabidopsis*, where an auxin minima is needed for vegetative axillary meristem initiation (Wang Q. et al., 2014; Wang Y. et al., 2014). LAX1 is shown to interact with LAX PANICLE2 (LAX2, encoding a novel nuclear protein), and their double mutant shows severe phenotype in reproductive and vegetative axillary meristem development (Tabuchi et al., 2011). Thus, *LAX1* functions in tiller and inflorescence meristem initiation independently or together with *LAX2* via regulating auxin signaling and transport.

The homolog of *Arabidopsis TOPLESS (TPL*) gene in rice is *ABERRANT SPIKELET AND PANICLE 1* (*ASP1*) that encodes a TPL-like transcriptional corepressor and regulates the panicle morphology. The *asp1* mutant displayed pleiotropic morphological abnormalities with around 80% reduction in rachis length with shorter primary branches and a reduction in the number of normal spikelets. In addition, the spikelet morphology was also severely affected. These characteristic phenotypes are due to aberrations in the fate of reproductive meristems, i.e., *asp1* fails to fine tune the proper initiation and maintenance of inflorescence meristem, branch meristem, and spikelet meristem in a stage-specific and time-dependent manner. On investigating the role of *asp1* in response to auxin, a marked upregulation in the expression of *OsIAA20* (a marker of auxin-dependent gene induction) in comparison to wild type was observed, suggesting that the auxin signaling was disrupted. Thus, *ASP1* upholds the auxin signaling by forming a repressor negative feedback complex with *OsIAA20* and thereby regulating panicle morphology (Yoshida et al., 2012) (Figure 2).

As discussed above, the combined effects of the core components of auxin machinery greatly affect the plant as well as panicle architecture. However, exploring the complexity of hormonal crosstalks will open up a broader network explaining each landmark events of panicle development (Figure 2).

### Strigolactones

**FIGURE 3:**
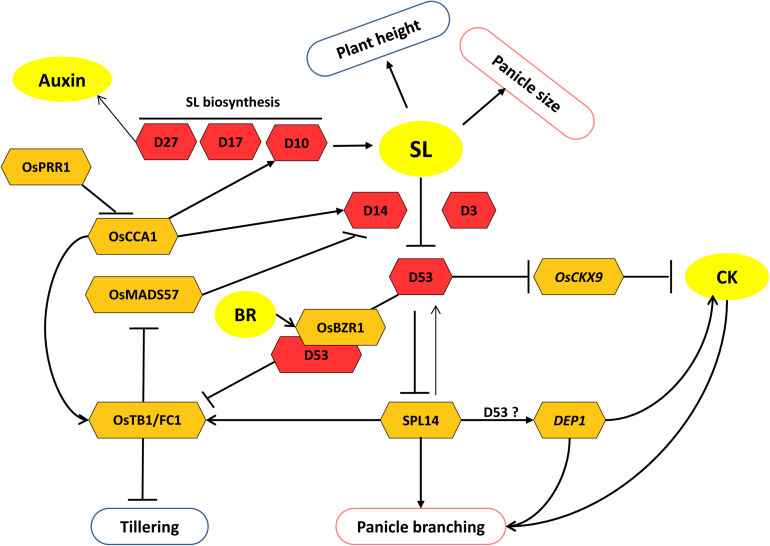
Model of strigolactone (SL) regulated pathways specifically involved in the regulation of panicle and tiller development. SL inhibits tillering but promotes panicle size and branching. Crosstalk of SL with BR and CK regulates tillering and panicle branching, respectively. Core components of SL biosynthesis and signaling are shown as red hexagons. Other interacting factors are shown as yellow hexagons. Arrows represent positive regulation; bar-headed lines represent negative regulation.

Strigolactones (SLs) are newly discovered carotenoid-derived plant hormones that play an inhibitory role in shoot branching in diverse species (Dun et al., 2009). In rice, mutant screening has revealed the involvement of various genes in SL biosynthesis and signaling pathways. Several of them control rice branching, which includes both shoot (tiller) and panicle branching (Kebrom et al., 2013). These mutants are marked by their short stature along with high tillering features. Hence, the genes involved in these mutants, i.e., *d3*, *d10*, *d14*, *d17* (or *htd1*), and *d27*, negatively regulate tiller bud activity in rice. Thus, they are named after their conspicuous dwarf phenotype as *DWARF* (*D*). Loss-of-function mutants of these genes show a subtle effect on panicle size and primary panicle branching. These mutants possess small panicles as compared to their respective wild types (Ishikawa et al., 2005; Zou et al., 2006). *D17*, *D10*, and *D27* genes are involved in the SL biosynthesis pathway, while *D3* and *D14* play an important role in SL signaling. *D17*/*HTD1 (HIGH-TILLERING DWARF1)* and *D10* encode CCD7 (CAROTENOID CLEAVAGE DIOXYGENASE 7) and CCD8, respectively (Arite et al., 2007; Umehara et al., 2008) (Figure 3). *D27* encodes a chloroplast localized iron-containing β -carotene isomerase enzyme that converts all *trans*-β-carotene into 9-*cis*-β-carotene, which is sequentially cleaved by the action of CCD7 and CCD8 to give SL precursors (Hao et al., 2009; Alder et al., 2012) (Figure 3).

*D14*/*D88*/*HTD2* encodes for the receptor for SL perception (Beveridge and Kyozuka, 2010) and negatively regulates the tiller bud outgrowth. Its loss of function results in a significant reduction in panicle size along with higher tillering, reduced height, and smaller seed size (Arite et al., 2009; Gao et al., 2009; Liu et al., 2009). Another loss-of-function allelic mutant of *D14*, *htd4*, bears a large number of effective panicles per plant. However, traits like panicle length, number of grains per panicle, primary branch numbers, seed-setting rate, and 1,000-grain weight are decreased in *htd4* plants (Wang et al., 2017). Recently, a new loss-of-function allele of *D14*, i.e., *dhta-34*, has been identified, whose plants also show similar phenotypes (Liang et al., 2019). Transcriptional regulation of *D14* has been shown to be mediated by OsMADS57. OsMADS57 negatively regulates *D14* expression and thus disrupts SL signaling and promotes tillering. However, its availability to regulate *D14* is limited by interaction with OsTB1 (TEOSINTE BRANCHED1) (Guo et al., 2013) (Figure 3). Although the role of *OsMADS57* in controlling panicle branching has not been observed, it affects panicle exsertion by regulating GA-deactivating genes (details in section “Gibberellic Acid”). *OsTB1*, also known as *FINE CULM1* (*FC1*), negatively regulates tillering in rice. It functions downstream of SL signaling and suppresses tillering by inhibiting the outgrowth of axillary buds (Minakuchi et al., 2010).

Other components involved in SL signaling are D3 and D53. D3 is an F-box protein that can make a complex with D14 (receptor) and D53 proteins. D53 is a molecular target of SL-induced degradation by a proteasome–ubiquitin pathway in a D14- and D3-dependent manner (Zhou et al., 2013) (Figure 3). It belongs to class I Clp ATPase proteins and contains a repression motif (EAR motif). In addition, its gain-of-function mutation results in small panicles, exaggerated tillering, and short stature. Thus, it acts as a repressor of the SL signaling pathway. Studies have suggested the involvement of D53 in the regulation of important panicle architecture governing genes. Like, in the absence of SL, D53 interacts with transcription factor SPL14/IPA1 (a key positive regulator of panicle branching) and corepressor TPL/TPR and inhibits IPA1 transcriptional activity. In the presence of SL, D53 undergoes proteasomal degradation, resulting in the release of repression of IPA1-regulated gene expression (Jiang et al., 2013; Song et al., 2017). Moreover, a negative feedback regulation exists because IPA1 directly binds to the promoter of *D53* and promotes its expression (Figure 3). *IPA1* expresses primarily during young panicle development. SPL14 promotes primary branch number in rice panicles that can result in increased grain productivity. SPL14 also promotes secondary branching in panicles (Xie et al., 2006; Jiao et al., 2010; Miura et al., 2010). Reports also indicate that SPL14 positively regulates the expression of *DEP1*, another important gene that determines panicle architecture and grain yield (Huang et al., 2009; Lu et al., 2013). However, it is still a matter of investigation whether D53 is also involved in the regulation of *DEP1* via SPL14 (Figure 3). Recently, D53 has also been shown to inhibit the expression of *OsTB1* gene. This suggests a possible way to regulate tillering negatively by SL signaling. SL perception by D14 causes D53 degradation and thereby promotes the expression of *FC1*, which negatively regulates tillering in rice (Fang et al., 2020). Besides, *OsTB1* also facilitates SL signaling by promoting the expression of *D14* by limiting the availability of its negative regulator, i.e., OsMADS57, by interacting with it (Guo et al., 2013). Moreover, SPL14 also regulates tillering through *OsTB1* in rice. It positively regulates the expression of *OsTB1* and suppresses tillering (Lu et al., 2013) (Figure 3).

A new finding has revealed the role of circadian clock in the regulation of traits like tillering and panicle development through the SL pathway (Strable, 2020; Wang F. et al., 2020). Two important clock regulators in rice, *CIRCADIAN CLOCK ASSOCIATED1* (*OsCCA1*) and *PSEUDORESPONSE REGULATOR1* (*OsPRR1*), are antagonistic factors of the clock component. *OsCCA1* mediates the rhythmic expression of the clock and output genes during plant growth and development, whereas *OsPRR1* negatively regulates the expression of *OsCCA1* (Wang F. et al., 2020). The study showed that increment and reduction in *OsCCA1* expression in transgenic plants decreased and increased the axillary tiller bud formation, respectively. Further, *OsCCA1*^*ox*^ plants had increased panicle size. Reverse phenotypes were observed for *OsPRR1* expression modified plants. The authors showed that OsCCA1 protein directly binds to the promoter of *D10*, *D14*, *OsTB1*, and *IPA1* and promotes their expression. D10 and D14 being SL biosynthetic enzyme and SL receptor, respectively, are effectors of SL concentration and perception. Thus, *OsCCA1* affects the SL pathway at both SL biosynthesis and signaling levels and negatively regulates tillering by repressing tiller-bud outgrowth and positively regulates panicle development by directly promoting the expression of *IPA1* (Wang F. et al., 2020) (Figure 3).

The exclusive role of SL in the regulation of panicle architecture has not been reported. Still, key regulators like SPL14 and DEP1, which control panicle branching and, thus, affect grain yield, seem to be regulated through SL signaling. Furthermore, the involvement of the SL pathway in the suppression of tiller development has been established. OsTB1 appears to be an integrator downstream of SL signaling that affects the suppression of tiller development. Taken together, it is evident that the SL pathway regulates both yield-determining traits, i.e., tiller and panicle development (Figure 3).

### Gibberellic Acid

Gibberellic acid (GA) is a well-known class of phytohormones involved in the regulation of various processes of plant growth and development. The outcomes of manipulations of GA levels or responses with respect to stem elongation have substantiated its potential for generating high-yielding cultivars in cereal crops. However, GA has also been reported to regulate other yield-related traits like panicle exsertion and panicle branching (Gao and Chu, 2020).

**FIGURE 4:**
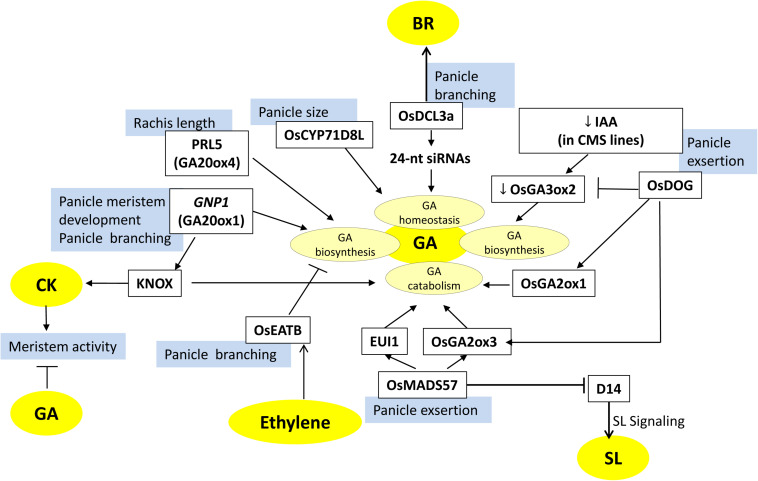
Factors involved in gibberellin (GA)-mediated regulation of panicle architecture in rice. The model depicts the factors (white boxes) that are involved in GA biosynthesis, catabolism, or homeostasis and influence panicle development and morphology. Blue boxes represent the regulated panicle phenotypes. Arrows represent positive regulation; bar-headed lines represent negative regulation. Cytokinin (CK) and GA have antagonistic roles in regulating reproductive meristem activity. GA modulating factors also interact with other hormones (BR, SL, ethylene) mediated factors to make a complex meshwork.

Studies have suggested that panicle exsertion length (PEL) is regulated in a GA-dependent manner. PEL is the length of the peduncle that emerges from the flag leaf sheath. In some rice varieties, shorter PEL leads to a situation of panicle enclosure, i.e., panicles are partly or fully enclosed within the flag leaf sheath. It is mainly caused by the shortening of the uppermost internode (UI). Panicle enclosure is usually a problem associated with the cytoplasmic male sterile (CMS) lines used in hybrid rice seed production. It blocks normal pollination in hybrid rice and thus reduces seed production. It has been observed that panicles of CMS lines are deficient in IAA levels, which causes downregulation of GA biosynthesis gene *OsGA3ox2*. This results in low levels of bioactive GA (GA_1_) in the UI leading to a reduction in the cell number and cell elongation. Therefore, UI is not long enough to push panicle out of flag leaf sheath, which leads to the panicle enclosure (Yin et al., 2007). Reverse phenotypes of increased panicle exsertion and UI elongation have been observed for loss-of-function *eui1* mutants. *EUI1* (*ELONGATED UPPERMOST INTERNODE1*) preferentially expresses in young panicles and encodes for a GA-deactivating enzyme (Luo et al., 2006; Zhu et al., 2006). Consequently, a higher bioactive GA level in the UI of *eui1* plants results in increased panicle exsertion. A recent report indicates that the expression of *EUI1* along with the expression of other GA deactivating gene *OsGA2ox3* (encodes GA 2-oxidase) is negatively regulated by the OsMADS57 transcription factor. Thereby, knockdown plants of *OsMADS57* contain low levels of bioactive GA due to elevated expression of *OsGA2ox3* and *EUI*, resulting in severe panicle enclosure and semi-dwarf phenotype (Chu et al., 2019). Another gene, *OsDOG* (*DWARF RICE WITH OVEREXPRESSION OF GIBBERELLIN-INDUCED GENE*), has been identified with a role in panicle exsertion, as its overexpression leads to a shorter PEL along with a dwarf phenotype. *OsDOG* (*or OsSAP11*) encodes A20/AN1 zinc-finger protein, which enhances the expression of GA catabolism related genes, whereas it downregulates the expression of GA biosynthesis gene (*GA3ox2*). Thus, unlike *MADS57*, *OsDOG* negatively regulates GA-mediated cell elongation and thus affects panicle exsertion (Vij and Tyagi, 2006; Liu et al., 2011) (Figure 4).

Gibberellic acid also affects other panicle-associated traits like panicle length, primary branching, secondary branching, and grain number per panicle. *OsCYP71D8L*, encoding a cytochrome P450 monooxygenase, controls panicle-related traits by affecting GA homeostasis. Gain-of-function of *OsCYP71D8L* leads to reduced panicle length, reduced grain number per panicle, and dwarfed plants (Zhou et al., 2020) (Figure 4). In a latest study, a gene, *Prl5* (*PANICLE RACHIS LENGTH5*), has been identified by QTL analysis by making a cross between two rice cultivars ST-1 and Koshihikari, which have distinct difference in panicle length. *Prl5* expresses mainly in young panicles and encodes a gibberellin biosynthesis enzyme, OsGA20ox4. It exclusively affects panicle architecture, as it promotes panicle rachis elongation and lowers primary branch elongation. Its expression in the vascular bundles, located near the lower primary branch meristem of young panicles, results in more accumulation of bioactive GA forms. This results in the elongation of both panicle rachis and lower primary branches of the panicle (Agata et al., 2020) (Figure 4).

These evidence clearly indicate the involvement of GA in the regulation of several panicle-related traits and show its potential to improve valuable agronomic traits in rice governing grain number per plant.

### Brassinosteroids

Brassinosteroids (BRs) are a class of novel, naturally occurring, plant-specific steroidal hormones, which are featured by their polyhydroxylated sterol structure and play critical roles in mediating multiple biological processes like development and stress response (Yang et al., 2011; Saini et al., 2015).

Two BR biosynthetic genes, *DWARF4 (D4) and DWARF11* (*D11*), encoding cytochrome P450 (CYP90B2 and CYP724B1, respectively) with redundant functions are implicated in regulating plant architecture (Tanabe et al., 2005; Sakamoto et al., 2006a). *PANICLE MORPHOLOGY MUTANT 1 (PMM1)*, *GRAIN NUMBER AND SIZE ON CHROMOSOME 4* (*GNS4*), *CLUSTERED PRIMARY BRANCH 1* (*CPB1*), and *NOTCHED BELLY GRAIN 4* (*NBG4*) are all different alleles of *D11* (Tanabe et al., 2005; Wu et al., 2016a; Zhou et al., 2017; Li et al., 2018; Tong et al., 2018). Gene insertional mutant library screening resulted in the identification of a mutant *pmm1-1*, which showed morphological defects of clustered branch phenotype, i.e., each panicle branch clustered with two to three abnormal spikelets (Li et al., 2018). Enhancing the expression of *D4* (role in C-22 hydroxylation, a rate-limiting step in BR biosynthesis) in the *pmm1-1* background rescued the abnormal inflorescence phenotype of *pmm1-1* mutant, indicating that BR deficit in the mutant was complemented by higher expression of *D4* (Sakamoto et al., 2006a; Li et al., 2018). Preferential expression of *PMM1* in developing young panicles, specifically in branches and spikelet primordia, established the role of *PMM1* in determining inflorescence architecture.

**FIGURE 5:**
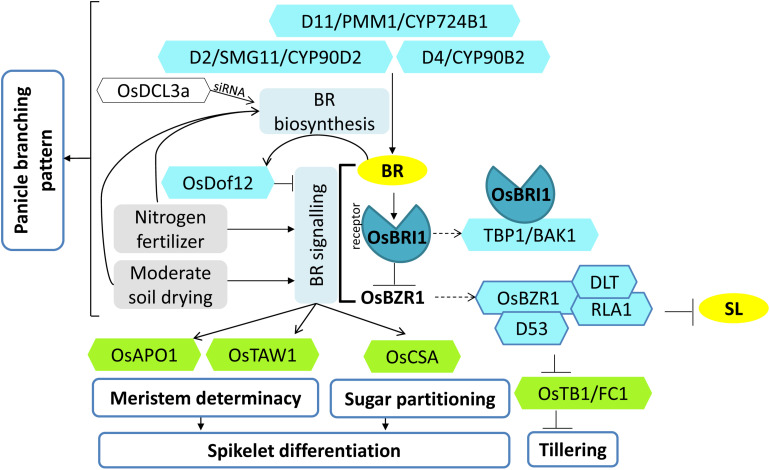
Model of brassinosteroid (BR) regulation controlling panicle development, panicle architecture, and tillering. Various factors (blue hexagons) regulate BR biosynthesis and signaling. Their coordinated actions regulate the downstream targets (green hexagons) of BR signaling that control various aspects of spikelet differentiation, panicle development, and tillering. Nitrogen and moderate soil drying conditions serve as additional factors affecting panicle development by increasing BR levels and signaling. Arrows represent positive regulation; bar-headed lines represent negative regulation; dotted arrows represent the formation of protein complex.

Mutant screening identified another regulator of grain size, grain number, and grain yield named, *SMALL GRAIN 11* (*SMG11*), which is a novel allele of *DWARF2* and encodes a cytochrome P450 (CYP90D2) involved in BR biosynthesis (Figure 5). The morphological traits of the mutant, *smg11*, include erect, shorter, and denser panicles at the mature stage, which is due to decreased length of rachis and an increase in the number of secondary branches in addition to smaller grains. Overexpression of *SMG11* using *ACTIN* promoter produced different lines with different levels of *SMG11* expression. Those with very high *SMG11* expression had a reduction in yield due to large and heavy seeds but reduced panicle branching. However, in lines with only a little increase in *SMG11* expression, plants had greater yield due to an increase in grain size and normal panicle branching. Thus, the levels of BR as a function of SMG11 can be optimized to improve the grain yield (Fang et al., 2016).

Moreover, *OsBAK1* (*BRI1-ASSOCIATED RECEPTOR KINASE*)/*TBP1* (*TOP BENDING PANICLE 1*), encoding a somatic embryogenesis receptor kinase (SERK) domain-containing protein involved in BR signaling also regulates grain number and grain size (Lin et al., 2017; Yuan et al., 2017). The *TBP1* mutation resulted in an increased seed number due to increased tillering and greater primary and secondary panicle branching. However, the panicle length and the seed size were reduced; hence, an overall reduction in yield was observed. TBP1 interaction with the BR receptor (OsBRI1) was reduced in the *tbp1* mutant (Figure 5). Only a limited overexpression of TBP1 increased the yield by obtaining a balance between grain size and grain number; on the other hand, greater overexpression reduced the yield drastically (Lin et al., 2017). Thus, BR signaling components cannot be altered to greater levels, but careful fine tuning can help in higher yields.

A Dof transcription factor, OsDof12, when overexpressed in rice, displayed altered plant architecture with reduced plant height and noticeable reduction in the number of primary branches, secondary branches, number of spikelets per panicle, and overall size of the panicle. BR levels were found to be the same in the wild type and the overexpressor. Thus, the BR metabolic genes remain unaffected; however, the transcript levels of two BR signaling genes, *OsBRI1* and *OsBZR1*, were downregulated. Further, *Dof12* itself gets upregulated by BR treatment (Wu et al., 2015). Hence, *OsDof12* acts as a negative regulator of BR signaling in rice wherein it is a BR responsive gene (Figure 5). Thus, OsDof12 may be regulating the BR signaling homeostasis to regulate panicle architecture via a negative regulatory feedback loop.

The final panicle size is contributed by spikelet differentiation and spikelet degeneration. The phenomenon of young spikelet degeneration at the basal region of the panicle, often referred to as “preflowering floret abortion,” causes severe yield loss in rice (Kato et al., 2008). The role of BR regulating spikelet abortion in young rice panicles has been recently studied wherein moderate soil drying treatments (soil water potential of -10 to -15 kPa) during panicle development enhanced BR biosynthesis in panicles. The higher expression level of *D11* led to increased BR contents [24-epiCS (24-epicastasterone) and 28-homoBL (28-homobrassinolide)] and ascorbic acid content in moderate drying-treated plants as compared to severe drying (soil water potential of -30 to -35 kPa) treated plants. Enhanced BR concentration suppressed the spikelet degeneration and enhanced spikelet differentiation, thus resulting in more spikelets per panicle. In addition, elevated expression of major determinants of spikelet meristem specification (*OsTAW1 and OsAPO2*), and sugar partitioning (*OsCSA*, a MYB domain protein), supports the notion that BR levels in young rice panicles promote spikelet differentiation condition by enhanced meristem activity as well as non-structural carbohydrate partitioning in moderate soil-drying treatment (Zhang et al., 2019a). Further, another study emphasized the role of BR in nitrogen-fertilizer-mediated enhancement of rice spikelet differentiation. Rice grown in different nitrogen fertilizer treatment, when supplied with exogenous BRs at spikelet primordium differentiation stage, resulted in elevated levels of endogenous BRs along with an upregulated expression of genes participating in BR biosynthesis (*OsD2*, *OsD11*) and BR signaling cascade (*OsBRI1*, *OsBAK1*) (Figure 5). These changes positively correlated with high H^+^ATPase activity, high ATP concentration, and high energy charge in panicles. These all together promoted spikelet differentiation and reduced spikelet degeneration (Zhang et al., 2019b).

The magnitude of actual crop productivity is determined by the level of spikelet sterility in rice. Hence, manipulating genes regulating BR biosynthesis and signaling during spikelet development will be a feasible approach for increasing grain yield (Figure 5).

### Ethylene

Ethylene is a gaseous phytohormone known to be coordinating a vast array of developmental processes in plants. The dynamics of ethylene action is due to the equilibrium between its biosynthesis and its perception. In terms of contribution to important agronomic traits in rice, ethylene has a role in the regulation of panicle architecture, grain size control, and grain filling rate (Yin et al., 2017).

Rice panicle has asynchronous grain filling, i.e., the degree and rate of grain filling in individual spikelets depend on its position in the panicle (Mohapatra and Sahu, 1991). The superior spikelets located in apical branches flower early and fill faster to produce larger and heavy grains. The inferior spikelets present in proximal branches fill slowly and lack quality due to poor filling, making it unsuitable for human consumption. The poor grain filling can be attributed to the higher evolution rate of ethylene in inferior spikelets. These spikelets show significantly higher expression of *S*-adenosyl methionine synthase (SAM-synthase), an enzyme that produces *S*-adenosyl methionine (precursor of ethylene biosynthesis) from methionine. This high evolution rate of ethylene greatly affects the rates of cell division in the endosperm and, thus, the grain filling rate. Application of 1-methylcyclopropene (1-MCP), a potent ethylene action inhibitor, shows a pronounced inhibitory effect on inferior spikelets. 1-MCP treatment enhanced endosperm growth at the mid-grain filling stage by facilitating cytokinesis and endo-reduplication, through enhancing the expression of several cell cycle regulators like cyclins, cyclin-dependent kinase (CDK), and cyclin-dependent kinase inhibitor (CKI) (Panda et al., 2016).

**FIGURE 6:**
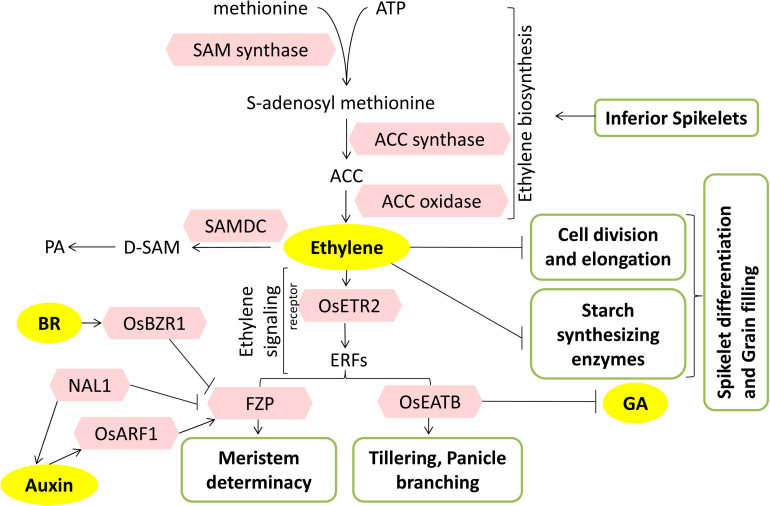
Mode of action of ethylene during panicle development and grain filling. The model summarizes the role of ethylene-related factors (pink hexagons) in the regulation of panicle development and grain filling. Inferior spikelets accumulate ethylene by promoting its biosynthesis. Polyamines compete and reroute ethylene metabolism, thus antagonizing the ethylene response. Meristem transition determination by FZP greatly affects the branching potential of panicles. FZP interacts with BR and auxin pathway components. OsEATB coordinately with GA affects the tillering and panicle branching. PA, polyamines; D-SAM, decarboxylated SAM; SAMDC, SAM decarboxylase. Arrows represent positive regulation; bar-headed lines represent negative regulation.

The biosynthetic pathways of ethylene and polyamines (PAs) are interrelated through a key branch point intermediate, SAM (Figure 6). An increase in PA biosynthesis through SAM decarboxylase (SAMDC) activity greatly affects the ethylene biosynthesis rate (Walden et al., 1997). The potential metabolic interaction or competition between the two affects the spikelet development under various degrees of soil drought. Moderate drought at the early endosperm cell division stage and grain filling stage significantly increases the free PAs (spermidine and spermine) levels, which lead to a decrease in ACC (1-aminocylo-propane-1-carboxylic acid, ethylene precursor) content as well as ethylene evolution rate (Zhang W. et al., 2017). Moreover, the application of PAs to young panicles also affected the activity of various enzymes (sucrose synthase, ADP glucose pyrophosphorylase, soluble starch synthase) involved in sucrose–starch metabolic pathway. This, in turn, led to a higher cell division rate in the endosperm, a higher percentage of filled grains, and increased grain yield (Yang et al., 2008). Ethylene also negatively regulates postanthesis spikelet development and grain filling in coordination with abscisic acid (ABA). The lower ratio of ABA to ethylene and ACC contents in inferior spikelets directly correlates with the rate of cell division and grain filling rate and hence grain weight (Yang et al., 2006).

Transgenic and mutant studies have also conveyed the role of ethylene in grain filling. Overexpression of *ETHYLENE RESPONSE2* (*ETR2)*, an ethylene receptor, showed a reduction in the effective number of panicles, lowered seed setting rate, delayed floral transition, and enhanced starch accumulation in internodes. Further, *etr2* mutants and *ETR*-RNAi plants had considerably higher 1,000-grain weight due to better grain filling. Upregulation of flowering-related genes, *GIGANTEA (OsGI)* and *TERMINAL FLOWER1/CENTRORADIALIS homolog (RCN1)*, in *ETR2*^*Ox*^ delayed the floral transition, whereas downregulation of *RAMY3D* (α-amylase gene) blocked sugar translocation from stems to grains leading to low seed setting rate and hence low yield (Hada et al., 2009) (Figure 6).

Ethylene response factors (ERFs) are transcription factors that are regulators of ethylene signaling and response. *FRIZZY PANICLE (FZP)*, an ERF transcription factor, is a rice ortholog of *BRANCHED SILKLESS1 (BD1)* in maize, having a key role in meristem transition and represses axillary meristem formation. The *fzp* mutant is characterized by the presence of sequential rounds of branching instead of florets. *FZP* is required to maintain the transition from spikelet meristem to floral meristem and to suppress the continued formation of axillary meristems from differentiated spikelet meristems (Komatsu et al., 2003). *FZP* regulates panicle branching by negatively regulating *APO2*/*RFL*, the gene responsible for regulating inflorescence meristem, and positively regulating several floral organ identity genes, especially the subset of B class (*OsMADS6*, *OsMADS17*) and E class (*OsMADS1*, *OsMADS7*, *OsMADS8*) (Bai et al., 2016). Recently, QTL *qSrn7* was identified, which is an allele of *FZP*. An increase in higher-order branching, especially in the upper regions or upper primary branches of panicles, was seen in lines carrying *qSrn7/FZP*. Thus, FZP regulates a unique branching pattern in rice, which is probably due to suppression of transition from branch meristem to spikelet meristem (Fujishiro et al., 2018) (Figure 6).

Investigating the changing concentrations of ethylene and dissecting the components of ethylene signaling machinery will be helpful in understanding the regulation of heterogeneous panicle architecture.

### Jasmonic Acids

A healthy spikelet formation not only determines the reproductive success in cereal crops but is also essential for obtaining better grain yield. Jasmonic acids (JAs) play a key role in this process, as several mutant studies have revealed the role of JA in spikelet morphogenesis. The mutants like *extra glume 1*, *eg1* (a recessive mutant of JA biosynthesis-related gene), and *eg2* (a dominant mutant of *OsJAZ1*) show defects in spikelet formation with increased glume like structures, altered floral organ identity, and floral organ number along with defects in floral meristem determinacy. JA signaling specifies these processes by regulating the E-class gene, *OsMADS1*, which is known to play a role in spikelet development (Cai et al., 2014). Moreover, spikelet development is also shown to be regulated by another gene, *OsPEX5*, which facilitates JA biosynthesis. OsPEX5 is a peroxisomal targeting sequence 1 (PTS1) receptor protein and helps in the import of an enzyme (OsOPR7) involved in JA biosynthesis into the peroxisome. Its loss of function results in abnormal spikelet morphology like extra glumes, abnormalities in the lemma and palea, formation of the lateral floret, and altered number/aberration in the stamen and pistil. More severe spikelet-related defects are associated with the *Osmyc2* mutant. OsMYC2 is an activator of JA-responsive genes that regulates the expression of some MADS genes like *OsMADS1*, *OsMADS7*, and *OsMADS14* and plays roles in spikelet development (You et al., 2019). Thus, abnormal spikelet morphogenesis resulting from altered JA biosynthesis/signaling can affect the grain yield. For example, the heterologous overexpression of *Arabidopsis JMT* (encodes jasmonic acid carboxyl methyltransferase enzyme that converts JA into methyl Jasmonate, MeJA) in rice results in the reduction in grain yield due to decreased number of spikelets per panicle and filling rates. Low grain yield is obtained due to altered spikelet development resulting from the increased levels (sixfold) of MeJA in young panicles. Furthermore, wild-type young panicles show much higher levels of MeJA (∼19-fold) under drought stress resulting in loss of grain yield (Kim et al., 2009). In addition, another JA biosynthesis mutant, *pre* (*precocious*), which primarily shows a defect in juvenile-to-adult transition, also possesses defect in seed set. In normal conditions, swelled lodicules result in flower opening, while their withering results in flower closing soon after flowering. However, *pre* plants bear non-withering lodicules that resulted in persistent flower opening, and thus, plants are mostly devoid of seed set. *PRE* encodes for allene oxide synthase (OsAOS1) enzyme involved in JA biosynthesis (Hibara et al., 2016). *NOG1* (*NUMBER OF GRAINS 1*) also affects JA biosynthesis and controls grain yield. *NOG1* encodes an enoyl-CoA hydratase/isomerase enzyme, which is involved in fatty acid β-oxidation pathway. Its higher expression results in the downregulation of the genes associated with the JA biosynthesis pathway leading to low endogenous JA levels. This results in the enhancement of grain number per panicle, and thus, grain number per plant is increased (Huo et al., 2017). These studies suggest the involvement of JA in spikelet development, which in turn plays a vital role in determining the grain yield.

### Crosstalk of Phytohormones to Regulate Grain Number

Signaling cascades and responses of several phytohormones are overlapping, and the molecular components are often shared among them. A complex network of effectors of multiple hormonal pathways collide and communicate to regulate critical agronomic traits. The following are a few well worked out examples reported so far.

A signaling module of *mitogen-activated protein kinases* (*MAPKs*), involving sequential phosphorylation of OsMKKK10-OsMKK4-OsMAPK6, is well established in regulating the seed size in rice by increasing cell proliferation (Xu et al., 2018). In this module, *OsMKK4 (smg1)* and *OsMAPK6* are known to regulate BR responses to affect grain size (Duan et al., 2014; Liu S. et al., 2015; Xu et al., 2018). Lately, a signaling link has been established between BR-mediated grain size regulation and CK-mediated grain number regulation in rice. *GRAIN SIZE AND NUMBER1* (*GSN1*) encodes another mitogen-activated protein kinase, OsMKP1, that regulates both grain size and grain number. The mutant *gsn1* had larger seeds but sparse panicle, whereas overexpression of *GSN1* results in an increased number of seeds per panicle but smaller seed size. Guo et al. (2018) showed that GSN1 negatively regulates the OsMKKK10–OsMKK4–OsMAPK6 cascade by directly dephosphorylating OsMAPK6/OsMPK6 (Figure 1). The CK levels were significantly reduced in *gsn1* with the increase in *OsCKX2* transcript and severe downregulation of CK-activating *LOG*. On the other hand, BR response was triggered in *gsn1* mutants by the upregulation of BR signaling genes (Guo et al., 2018). A recent study identified an upstream regulator, *ERECTA1* (*OsER1*), of the OsMKKK10–OsMKK4–OsMAPK6 cascade that encodes a receptor-like kinase protein. OsER1 activates the MAPK module either directly or indirectly, wherein OsMAPK6 directly interacts with DST to phosphorylate it (Figure 1). Phosphorylated DST transactivates the expression of *OsCKX2* that results in CK degradation. Interaction of GSN1 and OsMAPK6 maintains the homeostasis of CK levels (Guo et al., 2020) (Figure 1). Thus, CK and BR pathways contribute to a trade-off between grain number per panicle and grain size via MAP kinase signaling module (Figure 1).

Two recent studies have established that D53, a transcriptional repressor of the SL signaling, coordinates with components of SL, CK, and BR to regulate plant architecture that ultimately regulates grain number (Duan et al., 2019; Fang et al., 2020). In the first report, D53 has been shown to act as a link between SL and CK crosstalk, where D53 negatively regulates the expression of the CK catabolism gene, i.e., *OsCKX9* (*CYTOKININ OXIDASE/DEHYDROGENASE 9*) (Duan et al., 2019). *OsCKX9* encodes for a cytokinin oxidase enzyme involved in the degradation of CK, and it appears to be an SL-induced gene as a primary response of SL signaling. Its overexpression and loss of function display similar phenotypes of reduced panicle length, less primary and secondary branches per panicle, less grain number per panicle, along with other vegetative alterations like higher tillering and reduction in plant height. It has been evidenced that SL-induced degradation of D53 leads to an increase in the transcript and thus protein levels of *OsCKX9*, which catabolizes CK. In turn, low CK levels are responsible for reduced expression of a downstream CK-responsive gene *OsRR5* (type-A response regulator) (Duan et al., 2019). Therefore, SL regulates the rice branching, including panicle morphology, by activating CK catabolism (Figures 1, 3). In the second study, SL and BR signaling pathways coordinate and regulate tillering in rice. Fang et al. (2020) showed that D53 interacts and makes a complex with another transcriptional repressor BZR1, which functions downstream of BR signaling to regulate the expression of genes involved in tillering. The D53–OsBZR1 complex inhibits the expression of *FC1/TB1* gene that encodes a TCP transcription factor, which has been reported to inhibit shoot branching in rice and is a target of SL signaling (Takeda et al., 2003; Fang et al., 2020). OsBZR1 directly binds to the *FC1* promoter and recruits D53 to inhibit the expression of *FC1* in the tiller bud. The presence of SL induces degradation of D53–OsBZR1 complex and leads to the upregulation of *FC1/TB1* expression, and thus, SL signaling inhibits rice tillering. On the contrary, BR signaling leads to the accumulation of BZR1 as well as the downstream components of the BR signaling pathway, RLA1, and DLT. The accumulated OsBZR1–RLA1–DLT complex interacts with D53 and inhibits *FC1/TB1* expression and hence promotes tillering (Song et al., 2017; Fang et al., 2020). Thus, antagonistic regulation of D53–OsBZR1 complex stability by SL and BR regulates tillering (Figures 3, 5). SL and BR also regulate the panicle architecture independently, which raises the possibility of the existence of such coordination of SL and BR in controlling panicle-related traits also.

As another instance of crosstalk, two reports have shown that the upstream regulatory regions of ethylene-responsive factor *FZP*, which has a proven role in panicle branching, are occupied by BR and auxin-responsive transcription factors to regulate *FZP* expression. In the first report, a QTL named *Small Grain and Dense Panicle 7* (*SGPD7*) responsible for increased seed per panicle but decreased seed length was identified. *SGPD7* was identical to *FZP* with an 18-bp duplication in its ∼5.3 kb upstream region that turns out to be a silencer region. BR signaling transcriptional repressor, BZR1, was shown to bind to this region and represses the expression of *FZP* (Figure 6). The duplication of the silencer resulted in more number of secondary branches and more spikelets per panicle and an overall 15% increase in the yield (Bai et al., 2017). Second QTL, *CONTROL OF SECONDARY BRANCH 1* (*COS1*), also representing FZP, had a 4-bp deletion in its 2.7 kb upstream region. The deletion was shown to disrupt the binding of Auxin Response Factor6 (OsARF6) onto the FZP promoter and consequently results in decreased *FZP* expression, removal of branching repression, an increase in secondary branches, and more grain yield. Interestingly, this 4-bp deletion is shown to be strongly selected during domestication, as all 218 cultivated rice cultivars analyzed had this deletion (Huang et al., 2018). Moreover, FZP was shown to interact with NAL1 that encodes a trypsin-like serine and cysteine protease, which affects polar auxin transport. NAL1 negatively regulates FZP by enhancing its degradation (Huang et al., 2018) (Figure 6). Thus, BZR1- and OsARF6-mediated transcriptional control and NAL1-mediated posttranslational control of *FZP* makes a complex network of ethylene–BR–auxin pathways in regulating grain yield.

CKs and GAs play antagonistic roles in regulating reproductive meristem activity, with CKs having positive effects on meristem activity and maintenance, whereas GA is detrimental to meristem activity (Wu et al., 2016b). KNOTTED1-like homeobox (KNOX) proteins are homeodomain-containing transcription factors that accumulate in the cells around the meristem and regulate the accumulation of CK and GA in the meristematic cells by directly targeting their biosynthesis and catabolism genes, respectively. KNOX proteins upregulate the expression of members of the *OsIPT* gene family that increase CK biosynthesis intermediates and CK response (Figure 1). On the other hand, it lowers the GA levels by downregulating *GA20Oxs* genes that catalyze the GA biosynthetic steps for bioactive GA. Accordingly, *KNOX* genes establish a high CK and low GA level balance in the meristem to maintain its activity (Sakamoto et al., 2006b). The QTL, *GNP1* (*GRAIN NUMBER PER PANICLE*), encoding a GA biosynthetic protein, GA20Ox1, shows increased accumulation of *KNOX* transcripts and high inflorescence meristem activity. The upregulation of *GNP1* (using NIL-*GNP1*^*TQ*^) results in the increase in grain yield by 5.7–9.6% by enhancing the total grain number per panicle and secondary branching. Interestingly, NIL-*GNP1*^*TQ*^ plants contain a low level of bioactive GA, despite the fact that the expression of GA biosynthesis genes increases. *GNP1* increases CK activity in rice panicles via negative feedback of CK biosynthesis mediated by the *KNOX* gene. Simultaneously, it reduces GA accumulation in the panicles via upregulating GA catabolism genes (Wu et al., 2016b). Hence, the crosstalk between KNOX-mediated CK activity and GA activity regulates panicle architecture (Figures 1, 4).

Panicle development and branching are also regulated by the antagonistic interplay between CK and auxins. One of the core components of auxin signaling machinery is the F-BOX TRANSPORT INHIBITOR RESPONSE1/AUXIN SIGNALING F-BOX PROTEIN (TIR1/AFB) receptor that directly links auxin perception to the degradation of Aux/IAA proteins (Dharmasiri et al., 2005) (Figure 2). *Auxin-signaling F-Box 6* (*OsAFB6*) is preferentially expressed in the meristematic tissues (shoot apical meristem and young inflorescences), and its overexpression resulted in larger panicles with increased spikelets per panicle, more primary branch number, and a marked increase in yield by 50%. Assessment of endogenous hormone levels in the young panicles showed a drastic reduction in IAA levels, whereas the concentration of bioactive CKs was found to be significantly high in *OsAFB6*^*OX*^. Accordingly, the expression of *OsCKX2* was also downregulated in *OsAFB6*^*OX*^ (Figures 1, 2). Moreover, *WUSCHEL-LIKE HOMEOBOX (WOX3)*, which is known to positively regulate *KNOX* genes (promotes CK biosynthesis) via negative regulation of *YAB3*, was upregulated in *OsAFB6*^*OX*^ (Dai et al., 2007). Consequently, CK accumulated in young panicles of *OsAFB6*^*OX*^. Taken together, the opposite effects of CK and auxin in *OsAFB6*-mediated signaling provided robustness to the regulation of panicle development and branching and hence grain yield (He et al., 2018). Another report has shown that polar auxin transport required for axillary bud formation is affected by an SL biosynthetic enzyme, *Dwarf27* (*D27*), which encodes an iron-containing protein (Figures 2, 3). The mutation *d27* augmented the polar auxin transport and exhibited the phenotype-like increased tillering and reduced plant height. The *d27* phenotype was also rescued by SL application; thus, SL and auxins are shown to interact together to control shoot branching in rice (Hao et al., 2009).

The crosstalk between GA and ethylene also seems to influence panicle branching and tillering through *ethylene response factor associated with tillering and panicle branching* (*OsEATB*). It is an AP2/ERF family transcription factor that is downregulated by ethylene treatment and possibly a negative regulator of the ethylene signaling pathway (Qi et al., 2011). Contrary to a general ethylene response, overexpression *OsEATB* reduced the plant height and panicle length at maturity. Downregulation of *OsCPS2* (ent-kaurene synthase A) in *OsEATB*^*OX*^ transgenics restricted GA biosynthesis and decreased GA levels, leading to suppression in internode elongation and thus resulting in dwarf phenotype. Further, overexpression of OsEATB increased the number of panicles per plant, and spikelets per panicle, possibly because the reduction in plant height enhanced the branching potential of both tillers and panicles, thereby increasing yield. This intercommunication between GA and ethylene positively regulates the tillering and panicle branching in rice by a reduced responsiveness of GA during internode elongation via ethylene-induced decrease in GA biosynthesis (Qi et al., 2011) (Figures 4, 6).

An interesting report has shown that small RNAs also regulate the homeostasis of multiple hormones to coordinate the features of agricultural traits. *OsDCL3a* encodes an RNase III-class Dicer-like 3 enzyme involved in the production of 24-nt small interfering RNAs (siRNAs), especially from miniature inverted repeat transposable elements (MITEs). Downregulation of *DCL3a* expression resulted in smaller panicles and reduced panicle branching with severe effects on secondary branching. Furthermore, other traits like flag leaf angle and plant height were also affected. Reduction in *OsDCL3a* function upregulated the expression of GA and BR biogenesis-related genes, whereas it downregulated the expression of GA-deactivating gene (*EUI1*). Thus, OsDCL3a generated 24-nt siRNAs directly target the expression of GA and BR homeostasis-related genes and, thereby, affect panicle morphology and hence grain yield (Wei et al., 2014) (Figures 4, 5).

These interhormonal communications and coordinations are the core to the development of plant architecture. It is essential to understand them, to be able to fix the shortcomings of the available crop genotypes for the betterment of yield potential.

## Conclusion and Future Directions

Inflorescence architecture serves as a critical determinant of rice yield and contributes to securing reproductive success, and thus, it represents a major trait of agronomic importance. In cultivated varieties of rice, each spikelet is represented by a single floret and thus a single grain, in contrast to other grass crops. Increasing the number of florets per spikelet can be an innovative approach to increase the productivity of rice inflorescence. The terminal fertile floret is surrounded by lateral organs that are a pair of rudimentary glumes and a pair of sterile lemmas. Studies have suggested that sterile lemmas are rudimentary lateral florets, suggesting a three floret spikelet hypothesis in rice (Ren et al., 2020). Several reports have shown that hormonal signaling is a critical determinant of lateral organ development. OsMADS1 controls the determination and differentiation of lateral organs by stimulating auxin transport, signaling, and response. Conversely, it suppresses the CK response by directly repressing the A-type response regulators (ARRs) (Khanday et al., 2013). MULTIFLORET SPIKELET1 (MFS1) is an ERF family protein that determines lateral organ identity by regulating the expression of other vital determinants of lateral organs (Ren et al., 2013). *ASP1*, as shown to be regulating auxin signaling, also determines the identity of the lateral organs (Zhang T. et al., 2017). JA-mediated signaling is also evidently shown to regulate lateral organ identity. An understanding of genes in the conception of lateral florets and the knowledge of precise hormonal equilibrium required for its differentiation may eventually materialize the generation of fertile lateral florets.

Combining genes and QTL with a known positive effect on grain number is a logical strategy to enhance the grain yield. *GNP1* and *NAL1* both are implicated in positively regulating grain number per panicle by adjusting CK–GA homeostasis and polar auxin transport, respectively. Moreover, both have pleiotropic effects on the leaf size (source tissue). Introgression lines generated by combining *GNP1* and *NAL1* significantly enhanced the yield potential further, in comparison to each allele independently, by increasing the source strength (broader leaves) and sink capacity (more grains) (Wang et al., 2020b). Although the grain yield increased owing to increased grain number per panicle, the pleiotropic effects of decreased grain weight remained unavoidable (Wang et al., 2020b; Zhai et al., 2020). The yield potential of the plant can only be fully exploited if the coordination of source strength and sink capacities is achieved in harmony with the assimilate flow (Smith et al., 2018; Zhai et al., 2020). Acquiring the ability to break the linkage between grain number and grain size would be fundamental to increase grain yields credibly, and, for this, hormone dynamics is indispensable (Panigrahi et al., 2019).

Further, phytohormone pathways were exploited to manipulate plant architecture to improve yields in Green Revolution varieties (Wen et al., 2018). A recent report showed that semidwarf and increased lodging resistance phenotype contributed by the GA pathway gene *SD1* was strongly coselected with an allele of *HTD1/D17* that contributed SL-induced increment of tillers in elite high-yielding Green Revolution rice varieties. The pyramiding of the GA and SL pathways by artificial co-selection led to a beneficial change in plant architecture during rice Green Revolution (Wang et al., 2020a). Another significant study demonstrated the fine tuning of panicle architecture by QTL pyramiding (Agata et al., 2020). QTL *Prl5* (encoding a GA biosynthetic enzyme) and QTL *PRIMARY BRANCH LENGTH6* (*Pbl6*) were shown to regulate non-overlapping components of panicle architecture and function independent of each other. *Pbl6/ABERRANT PANICLE ORGANIZATION1* (*APO1*) encoding an F-box-containing protein regulates primary branching and length of the upper primary branch. *Prl5*, on the other hand, governs the length of lower primary branches and rachis length. A combination of *Prl5* and *Pbl6* resulted in longer and more branched panicles with spatially arranged grains due to long primary branches, thus higher yield. Agata et al. (2020) showed that a range of panicle architecture patterns could be generated by regulating the expression of *Prl5* and *Pbl6* without a tradeoff relationship. These diversities of panicle architecture can be of great use to the breeders.

Clues from other plant systems are indispensable in understanding the dynamics of phytohormones in plants. The added information can be transferred or borrowed to the rice system for yield management strategies. A study in *Arabidopsis* showed that, in addition to the quantity of CK, the quality of CK also governs the physiological function in regulating shoot growth. The two bioactive forms of CK, viz., iP-type [*N*^6^-(Δ^2^-isopentenyl) adenine] and tZ-type (trans Zeatin), differ in their side-chain moieties. The iP-type CK can be converted to tZ type by trans-hydroxylation by specific cytochrome P450s. A decline in this enzymatic activity decreases the shoot growth without any quantitative reduction in CK levels (Gao et al., 2019). It would be of interest to find how far this mechanism affects the shoot and inflorescence branching in rice. A recent report showed that a maize serine/threonine-protein kinase, encoded by *KERNEL NUMBER PER ROW6* (*KNR6*), positively regulates grain yield. *KNR6* is involved in inflorescence meristem development via phosphorylation of an ARF GTPase-activating protein (AGAP) (Jia et al., 2020). Rice OsAGAP is well implicated in auxin transport and localization in root development (Zhuang et al., 2006), but its function is not established in inflorescence meristem maintenance. Moreover, the orthologous gene for *KNR6* is not yet characterized in rice. Assessing the function of *OsAGAP* and *OsKNR6* and their interaction in inflorescence development via an auxin-mediated pathway in rice can be a future endeavor.

Hormonal networks are complex, interlinked, and regulated at multiple levels. However, the existing understanding of molecular resources for hormonal engineering has tremendous potential for manipulation of plant architecture for crop yield improvement and, at the same time, have pleiotropic effects. Hence, mindful pyramiding of genes with an understanding of cross-hormone signaling dynamics can plausibly tap the plant’s resources fully.

## Author Contributions

PD, AP, SS, and AKT compiled the literature, prepared the figures, and wrote the manuscript. AKT and PD conceptualized and designed the structure of the manuscript. All the authors read and approved the final manuscript.

## Conflict of Interest

The authors declare that the research was conducted in the absence of any commercial or financial relationships that could be construed as a potential conflict of interest.
